# Panoramic comparison between NK cells in healthy and cancerous liver through single-cell RNA sequencing

**DOI:** 10.20892/j.issn.2095-3941.2022.0050

**Published:** 2022-07-21

**Authors:** Huan Liu, Ronghua Zhao, Rongrong Qin, Haoyu Sun, Qiang Huang, Lianxin Liu, Zhigang Tian, Björn Nashan, Cheng Sun, Rui Sun

**Affiliations:** 1 The First Affiliated Hospital of USTC, Division of Life Sciences and Medicine, University of Science and Technology of China, Hefei 230001, China; 2 The CAS Key Laboratory of Innate Immunity and Chronic Disease, School of Basic Medical Sciences, Division of Life Sciences and Medicine, University of Science and Technology of China, Hefei 230027, China; 3 Institute of Immunology, University of Science and Technology of China, Hefei 230027, China

**Keywords:** Hepatocellular carcinoma, natural killer cell, single-cell RNA sequencing, heterogeneity, prognosis

## Abstract

**Objective:**

NK cells play crucial roles in the immune defense mechanisms against viral infections and transformed cells. However, the developmental progression, transcriptomic landscape, and functional subtypes of liver NK cells are not well defined. Hepatocellular carcinoma (HCC) accounts for approximately 80% of primary liver cancer worldwide, yet the biological characteristics of NK cells in the HCC environment are unclear. Therefore, we aimed to determine these cells’ roles in tumorigenesis and prognosis.

**Methods:**

We compared the single-cell RNA sequencing profiles of NK cells purified from blood (*n* = 1), healthy liver tissues (*n* = 3), HCC tumor tissues (*n* = 4), and peritumor liver tissues (*n* = 1) to identify NK cell subsets. Furthermore, we performed bioinformatics analysis by using The Cancer Genome Atlas (TCGA) data to identify prognostic biomarkers simultaneously overexpressed in the blood and tumor tissues of patients with HCC.

**Results:**

Transcriptomic analysis revealed 5 NK cell subsets (L1-NK-CD56^bright^, L2-NK-CD56^dim^, L3-NK-HLA, L4-LrNK-FCGR3A, and L5-LrNK-XCL1) in the healthy liver tissues. However, the transitional L3 subset and the CXCR6^+^CD16^+^ L4 subset with strong anti-tumor activity were absent in the HCC and peritumor liver tissues. Furthermore, 4 common prognosis-associated genes (*RHOB*, *TALDO1*, *HLA-DPA1*, and *TKT*) were significantly overexpressed in the paired tumor tissue and blood.

**Conclusions:**

Our study revealed 5 specific subsets of NK cells in healthy human liver tissues. However, only 3 of the 5 NK cell subsets were present in HCC and peritumor tissues. The cytotoxic NK cell subsets were absent in HCC tissues. Furthermore, we identified 4 potential non-invasive prognostic biomarkers in patients with HCC.

## Introduction

Natural killer (NK) cells and type 1 innate lymphoid cells (ILC1s) are different subsets of innate lymphoid cells (ILCs), according to the International Union of Immunological Societies, and these cells show significant differences in their development trajectories^1^. NK cells play major roles in the immune defense mechanisms against viral infections^2^ and cancer cells through cytolytic activity and the production of inflammatory cytokines^3^. They directly kill tumor cells that lack MHC class I molecules and enhance the functions of cytotoxic T lymphocytes^4^. Furthermore, NK cell-based immunotherapy has shown immense potential in the treatment of hematological cancers and solid tumors^5–8^.

In the peripheral blood, NK cells constitute nearly 10%–15% of all lymphocytes^9^. The venous blood is a common source of human NK cells for research studies. Human peripheral blood NK cells are classified into 2 subsets according to the surface expression of CD56 and CD16: the CD56^dim^CD16^+^ subset, which is highly cytotoxic, and the CD56^bright^CD16^-^ subset, which secretes specific cytokines^10^. A large NK cell population is present in the liver^11^, and it accounts for nearly 30%–50% of the hepatic lymphocytes^12^. In the liver, NK cells play important roles in liver tolerance and immunity^13^. The human liver hosts diverse liver-resident and conventional NK (cNK) cell populations. Although the murine liver-resident NK (LrNK) cells (CD49a^+^DX5^−^) have been described previously^14^, the phenotype of human LrNK cells remains obscure. Currently, human LrNK cells are identified according to the expression levels of the transcription factors Eomes, T-bet, and Hobit (Eomes^hi^T-bet^lo^Hobit^+^)^15–17^; the expression of cell surface markers such as CXCR6, CD69, and TIGIT; and the absence of CD49e expression on their cell surfaces^18,19^. However, the developmental progression, transcriptomic landscape, and heterogeneity of human liver NK cells is not well defined. Therefore, in-depth characterization of the liver NK cells is critical for understanding liver immunology under normal and cancerous conditions, and may aid in determining treatment strategies for patients with liver cancer.

Single-cell RNA sequencing (scRNA-seq) technology is a powerful tool to explore rare and heterogeneous cell populations^20–22^. A previous study has described a “transitional NK” subset from the scRNA-seq analysis of peripheral blood and bone marrow samples that represents an intermediate stage between CD56^bright^ and CD56^dim^ NK cells, according to the transcriptional profiles of the signature genes defining these 2 NK cell subsets^23^. Another scRNA-seq study on healthy human blood has demonstrated 3 new subsets of NK cells: type I interferon–responding CD56^neg^ NK cells, cytokine-induced memory-like NK cells, and NK cells with low ribosomal expression^24^. Furthermore, high-dimensional scRNA-seq analysis has demonstrated the conserved organ-specific features of the splenic NK cells in both humans and mice^25^.

Hepatocellular carcinoma (HCC) accounts for approximately 80% of all primary liver cancer cases worldwide^26^ and is the fourth most common cause of cancer-associated deaths globally^27,28^. The roles of different NK cell subsets may be critical for the development and progression of HCC. However, a previous scRNA-seq analysis of healthy human liver cells has identified NK cells in only 2 of 5 donors, because a mixture of parenchymal and non-parenchymal cells was present^29^. Furthermore, scRNA-seq analysis of liver-resident immune cells has not clearly identified distinct immunological characteristics of NK cell populations^30^. The comprehensive analysis of immune cell profiles provides a panoramic overall view but not extensive details regarding immune cell types. Furthermore, most scRNA-seq studies on immune cells derived from HCC tissues have focused on the characteristics of T cells, whereas the characteristics of NK cells have not been defined^31,32^. Hence, an urgent need exists to perform scRNA-sequencing analysis on NK cells derived from healthy liver and HCC tissues to discover new NK cell subsets that may be beneficial for immunotherapy. Therefore, in this study, we performed single cell transcriptome analysis of the NK cells from the liver, spleen, and peripheral blood of healthy control participants and patients with HCC to determine different subsets of liver-resident NK cells in healthy liver tissues and HCC tissues. We also analyzed potential prognostic genes in the NK cells isolated from paired peripheral blood and HCC tissues from a single patient with HCC.

## Materials and methods

### Human sample collection and ethics statement

The Ethics Committee of University of Science and Technology of China (2019 KY Ethics No. 116) and the Ethics Committee of the First Affiliated Hospital of University of Science and Technology of China [2019-N(H)-121] approved this study. All patients in this study provided written informed consent to sample collection and data analysis. The tumor tissues and peripheral blood were obtained from patients diagnosed with HCC on the basis of histopathology. Healthy liver tissues were obtained from participants without liver tumors. The clinicopathological characteristics of patients enrolled in cohort 1 are listed in **Supplementary Table S1**. Further details regarding the patients in cohort 1 are listed in **Supplementary Table S2**.

### Preparation of human lymphocytes

Fresh liver tissues (HCC or healthy liver) were minced into pieces. The tissue homogenates were treated with 1 mg/ml collagenase IV (Sigma) and 150 U/ml DNase I (Sigma) for 1 h. The digested tissue homogenates were then filtered through a 70-μm filter. Tumor infiltrating lymphocytes were then isolated through Percoll-Paque density centrifugation, and RBCs were removed through RBC lysis. Peripheral blood mononuclear cells were isolated with Ficoll-Paque density centrifugation. The lymphocytes were cryopreserved in 10% DMSO in FBS for further experiments.

### Cell sorting and flow cytometry

Briefly, individual human lymphocyte samples were thawed, washed, and incubated for 30 min at 4 °C in 1× PBS with 10% normal mouse serum (Beijing Yaanda Biotechnology). The cells were then stained with antibodies for 30 min at 4 °C in the dark. Then the cells were washed with MACS buffer and incubated with the dead cell marker 7-AAD. A FACSAria cell sorter (BD) was used to purify ILCs[Lin^-^ (CD34^-^CD3^-^CD14^-^CD1α^-^CD19^-^FcεRIα^-^) CD45^+^]. And a FACSCelesta instrument (BD) was used to detect NK cells. The details regarding the antibodies are shown in the Supplementary materials and methods (**Doc S1**).

### Single-cell RNA sequencing

The FACS-sorted ILCs were prepared for 10x Genomics scRNA-seq with the recommended sample preparation protocol, kept on ice, and counted. The intratumor and peritumor liver and blood samples were prepared according to the 10x Genomics Single Cell Reagent Kit user guide. RNA sequencing was performed by Genergy (Shanghai, China) on a Novaseq 6000 machine with a sequencing depth of at least 25,000 reads per cell. The data analysis methods used in this study are listed in the Supplementary materials and methods (**Doc S1**).

### The Cancer Genome Atlas (TCGA) data analysis

HCC patient (cohort 2 and cohort 3) data from TCGA database were used to evaluate the correlation between the selected genes and the survival of patients with HCC. The gene expression data and clinical data for patients with HCC were downloaded from cBioportal (https://www.cbioportal.org/).

### Statistical analysis

Statistical analysis was performed in GraphPad PRISM 6 software (GraphPad 6 Software). A *P* value < 0.05 was considered statistically significant (*P < 0.05, ** *P* < 0.01, ****P < 0.0001; ns, not significant). Bioinformatics data were statistically analyzed with R language. The bioinformatics analysis software and algorithms used in this study are listed in the Supplementary materials and methods (**Doc S1**).

## Results

### Transcriptome profiles of human NK cells significantly differ among the liver, spleen, and peripheral blood

**Figure 1:**
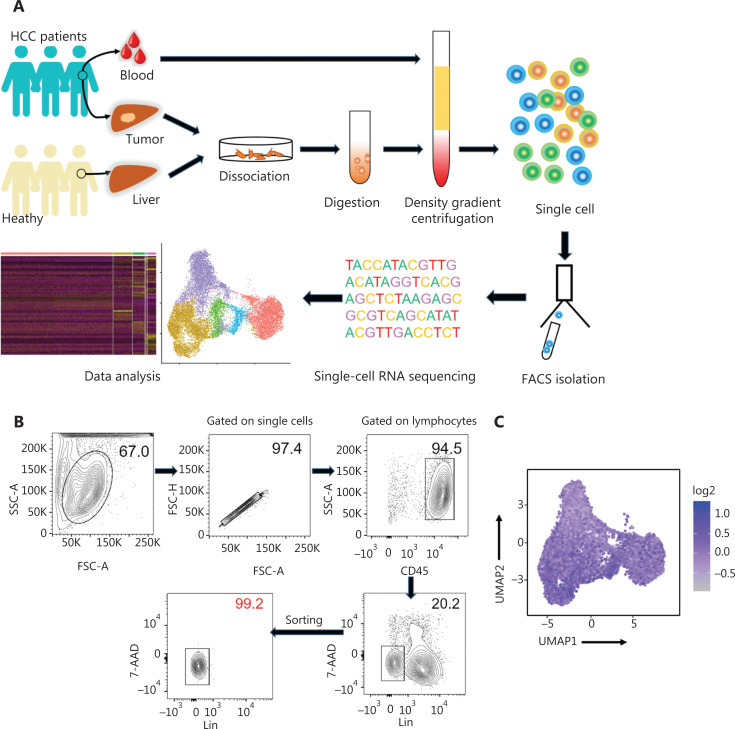
Study design and basic information for single cell RNA-seq analysis of human innate lymphoid cells. (A) Scheme of the overall study design. We performed 10x Genomics scRNA-seq on innate lymphoid cells derived from the livers (*n* = 3) of healthy control donors, as well as blood (*n* = 1), tumor (*n* = 4), and tumor-adjacent liver tissue (*n* = 1) from treatment-naive patients with HCC. The output data were integrated and combined for downstream analysis. (B) Flow cytometry plot of a representative human liver sample, illustrating the gating strategy used to sort innate lymphoid cells. Human innate lymphoid cells were defined as living single-cell Lin^-^ (CD34^-^ CD3^-^ CD14^-^ CD1α^-^ CD19^-^ FcεRIα^-^) CD45^+^ 7-AAD^-^ lymphocytes. SSC, side scatter; FSC, forward scatter. (C) UMAP plot showing NK cell feature scores. Feature genes, defined according to Crinier et al.^25^, included *CD160*, *CD244*, *CHST12*, *CST7*, *GNLY*, *IL18RAP*, *IL2RB*, *KLRC1*, *KLRC3*, *KLRD1*, *KLRF1*, *PRF1*, and *XCL2*.

High-throughput scRNA-seq was performed to investigate the biological roles of NK cells in the human liver. The human liver ILCs were sorted by flow cytometry from untreated patients with HCC (*n* = 4) and healthy control donors (HC, *n* = 3). Furthermore, fresh lymphocytes were isolated from paired intratumor (IT) tissues, peritumor (PT) tissues, and peripheral blood from a single patient with HCC (patient number 101) to compare the differences between local and peripheral sources in 1 person (**Figure 1A**). The purity of FACS-sorted Lin^-^ (CD34^-^CD3^-^CD14^-^CD1α^-^CD19^-^FcεRIα^-^) CD45^+^ ILCs exceeded 99% (**Figure 1B**). ILCs derived from the livers of HC donors (*n* = 3; HC1: 14,847 cells; HC2: 17,880 cells; and HC3: 16, 978 cells), peripheral blood (*n* = 1; 5,452 cells), HCC tumor tissues (*n* = 4; HCC1: 18,140 cells; HCC2: 10,264 cells; HCC3: 16,030 cells; IT: 10,771 cells), and peritumor liver tissues (*n* = 1; 10,135 cells) from treatment-naive patients with HCC were subjected to scRNA-sec with 10x Genomic technology. The 10x Genomics Cell Ranger software was used to process the sequences of 120,497 single cells with 29,385 mean reads per cell and 1,151 median genes per cell (**Supplementary Table S3**). The downstream data analysis was performed with the Seurat R package^33,34^, and the cells with low quality (number of genes less than 500 or more than 2,500) and a high (more than 5%) mitochondrial genome transcript ratio were removed. Furthermore, ILC1 (*IL7R* and *CXCR3*), ILC2 [*IL7R* and *PTGDR2* (*CRTH2*)], and ILC3 [*IL7R* and *KIT* (*CD117*)] cells were removed from the analysis on the basis of their maker genes, and only NK cells were selected for the final analysis, according to high expression of *NKG7* and the absence of *IL7R*^35,36^. The final analysis included 46,726 NK cell transcriptomes, including those from the blood (*n* = 1; 4,756 cells) and liver tissues from HC donors (*n* = 3; HC1: 6,234 cells; HC2: 2,124 cells; and HC3: 5,846 cells), as well as peritumor liver tissues (*n* = 1; 8,280 cells) and HCC specimens (*n* = 4; HCC1: 3,902 cells; HCC2: 4,799 cells; HCC3: 3,809 cells; IT: 6,976 cells). NK cell identity was confirmed according to the transcriptional status of *CD160*, *CD244*, *CHST12*, *CST7*, *GNLY*, *IL18RAP*, *IL2RB*, *KLRC1*, *KLRC3*, *CD94* (*KLRD1*), *NKp80* (*KLRF1*), *PRF1*, and *XCL2*^25^ (**Figure 1C**).

**Figure 2:**
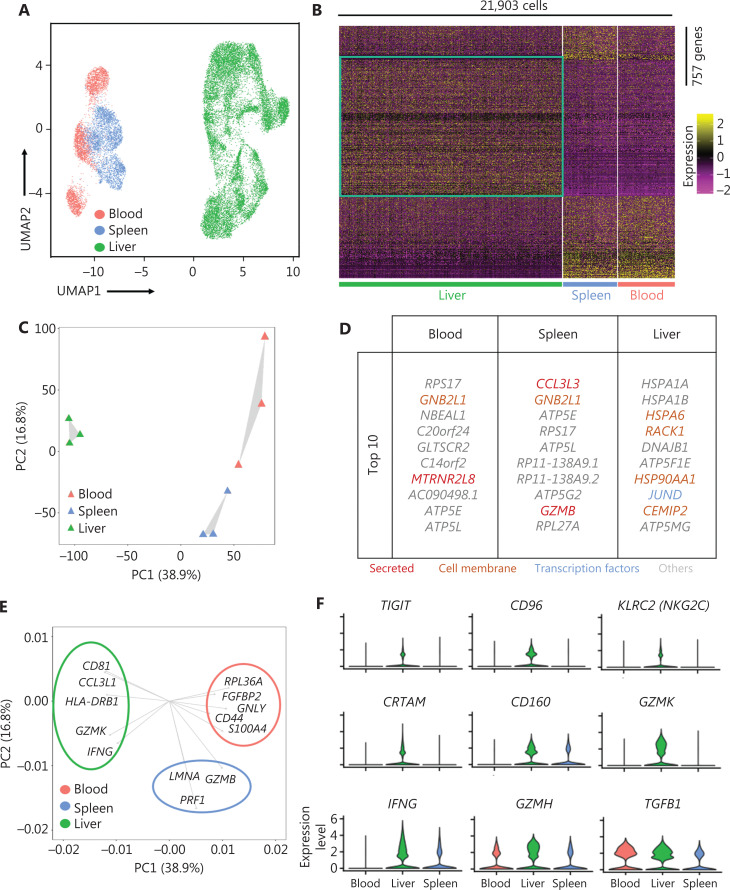
Liver NK cells are highly distinct from spleen and peripheral blood NK cells. (A) UMAP plot of healthy donor NK cells from human blood (red, 3,763 cells), spleen (blue, 3,543 cells), and liver (green, 14,597 cells) samples. (B) Heatmap of 757 informative genes for distinguishing human blood (213 genes), spleen (189 genes), and liver (355 genes) NK cells. Cells are plotted in columns by organ source, and genes are shown in rows, ranked by adjusted *P* value < 0.05 (Wilcoxon rank sum test). Gene expression is color coded with a scale based on z-score distribution, from −2 (purple) to 2 (yellow). (C) PCA for the 3 human organ NK cell subsets from each sample, according to the mean expression of the genes. Each triangle represents 1 sample, colored by organ. (D) Top 10 genes significantly differentially expressed among the 3 human organ NK cell subsets. Genes are ranked by log_2_ fold-change. (E) Genes driving human blood, spleen, or liver NK cell identity (according to the loading value), colored by cell origin. (F) Expression distribution (violin plots) in each population (horizontal axes), for known NK cell genes.

We investigated the heterogeneity of the human liver NK cells by comparing 3 liver, spleen^25^, and blood^25^ samples from healthy donors in the GEO database (GSE119562). The transcriptomic profiles of the NK cells were visualized with Uniform Manifold Approximation and Projection (UMAP) plots, in which each dot represented a single cell^37^. UMAP analysis of the blood (3,763), spleen (3,543), and liver (14,597) NK cells showed distinct differences in transcriptome characteristics (**Figure 2A**). We identified 757 genes (355 liver-specific, 189 spleen-specific, and 213 blood-specific) with significant differences in expression levels among NK cells derived from the liver, spleen, and blood (**Figure 2B**). The heatmap of gene expression showed a clear separation between liver NK cells and spleen and blood NK cells, according to the expression levels of these 757 genes (**Figure 2B**). Principal component analysis (PCA) also segregated the NK cells from the liver, spleen, and blood. PC2 separated the NK cells from those in the blood and the spleen; moreover, both PC1 and PC2 separated the liver NK cells from the blood and spleen NK cells (**Figure 2C**). These results suggested that liver NK cells had significantly different transcriptome characteristics from blood and spleen NK cells. Furthermore, these results suggested that the blood and spleen NK cells were more closely associated with each other than liver NK cells. The top 10 genes differentially expressed among the NK cells from the liver, blood, and spleen are shown in **Figure 2D**. We then confirmed the driver genes by mapping the genes in different subsets. *GZMB* and *PRF1* (targeted cell death-associated genes) were driver genes for splenic NK cells; *GNLY* (a cytotoxicity-associated gene) and *CD44* (a cell–cell adhesion and migration associated gene) were driver genes for blood NK cells; and *HLA-DRB1* (antigen presentation gene) and *IFNG* (soluble cytokine gene) were driver genes for liver NK cells (**Figure 2E**). Violin plot analysis indicated higher gene expression of inhibitory receptors such as *TIGIT* and *CD96*; activating receptors such as *KLRC2*, *CRTAM*, and *CD160*; cytolytic proteins such as *GZMK* and *GZMH*; and cytokines such as *IFNG* and *TGFB1* in NK cells from the liver than from the blood and spleen (**Figure 2F**). Together, these results demonstrated significant differences in the transcriptome profiles between the human liver NK cells and the NK cells from the spleen and peripheral blood.

### Unsupervised clustering of NK cells from the liver tissues of healthy controls reveals 5 distinct subsets

**Figure 3:**
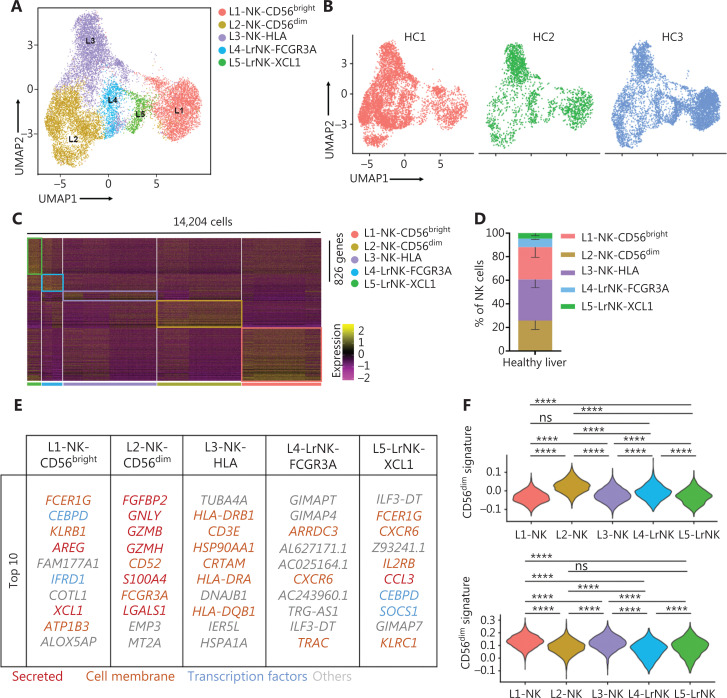

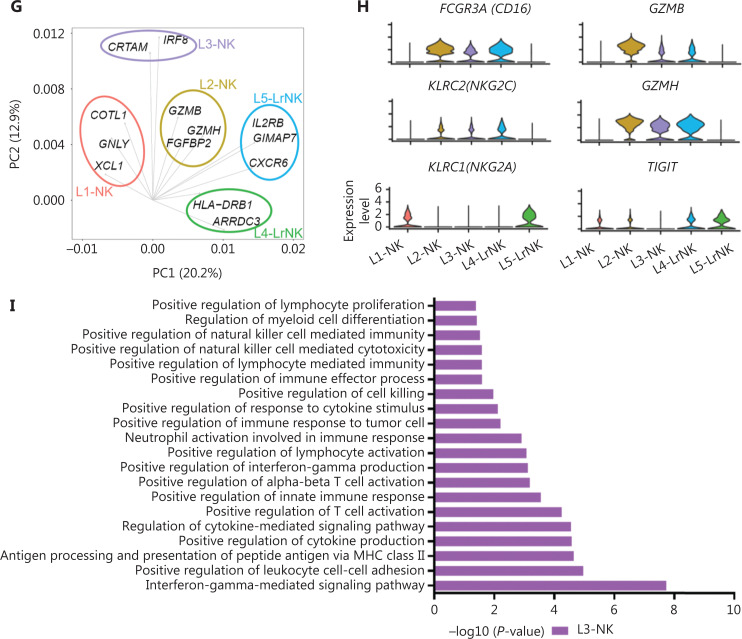
High-throughput scRNA-seq identifies 5 NK cell subsets in the healthy human liver. (A) UMAP projection of NK cells from an integrated analysis of NK cells from 3 healthy control donors, showing the formation of 5 main subsets identified according to the gene expression characteristics of each subset. (B) UMAP plot of human liver NK cells from healthy livers, colored by donor (HC1: 6,234 cells; HC2: 2,124 cells; and HC3: 5,846 cells). (C) Heatmap of the 826 genes assessed with a Wilcoxon rank sum test separating the 14,204 NK cells into 5 main subsets, shown in different colors. Squares identify specific transcriptomic signatures of the distinct cell subsets. (D) The proportions for the 5 subsets defined among NK cells from the 3 healthy control donors across all liver NK cells. (E) Top 10 genes significantly differentially expressed (Wilcoxon rank sum test) among the 5 liver NK cell subsets. Genes are ranked by log_2_ fold-change. (F) Module scores of CD56^dim^ and CD56^bright^ gene expression programs defined by Hanna et al.^43^. Violin plots representing the distribution of module scores for CD56^dim^ (top) CD56^bright^ (bottom) for each liver NK cell subset (Kruskal-Wallis ANOVA followed by Dunn’s multiple comparisons test). Nonsignificant (ns) *P* > 0.05; ****P < 0.0001. (G) PCA for the 5 liver NK cell subsets’ driving genes. (H) Violin plots comparing the expression of selected NK cell markers in this scRNA-seq dataset. The violin represents the probability density at each value. (I) Selected Gene Ontology terms using genes upregulated (log_2_ fold-change > 0.25) within the L3 subset with an adjusted *P* < 0.05.

Next, we integrated the scRNA-seq datasets of 3 HC donors by using the Harmony algorithm^38^ to identify the functional subtypes of NK cells in the human liver. The results revealed 5 different subsets (L1–L5) in the combined dataset (**Figure 3A**). Each subset consisted of cells from each donor, thereby indicating a well-integrated dataset (**Figure 3B**). The subset-specific transcriptomic signatures of each subset are represented by the heatmap in **Figure 3C**. The percentages of typical CD56^bright^ NK and CD56^dim^ NK subsets were similar, whereas the L3 subset represented the largest proportion of NK cells among the NK cell subsets in the healthy liver (**Figure 3D**). The top 10 differentially expressed genes among the 5 NK cell subsets are shown in **Figure 3E**. The L1, L2, and L3 subsets were identified as cNK cells according to their gene transcription signatures, whereas the L4 and L5 subsets were identified as LrNK cells on the basis of the expression of *CXCR6* and *EOMES* (**Supplementary Figure S1A and S1B**). The L1 subset (CD56^bright^ NK cells) displayed high expression of genes encoding soluble factors, such as amphiregulin (*ARGE*), *XCL1*, and *XCL2*, which have previously been used to identify the human CD56^bright^ NK cluster^23,39^. The L2 subset represented the CD56^dim^ NK cell cluster and showed significantly high expression of cytotoxicity-associated genes, such as *GNLY*, *GZMB*, and *GZMH*, in addition to overexpression of *FCGR3A* (encoding CD16), a typical feature of CD56^dim^ NK cells (**Figure 3E**). The L3-NK-HLA cluster showed moderate expression of *XCL1* and *FCGR3A* (**Supplementary Figure S1A and S1B**), and represented an intermediate phenotype between those of CD56^bright^ and CD56^dim^ NK cells, in agreement with findings from previous reports^10,40^. The L3-NK-HLA cells were characterized by high expression of *CRTAM* (activating receptor) and the human leukocyte antigens *HLA-DRB1* and *HLA-DRA*, which are associated with degranulation, proliferation, and IFN-γ secretion^41,42^. The L4-LrNK-FCGR3A and L5-LrNK-XCL1 cells showed high expression of *CXCR6* and *EOMES* (transcription factor) (**Figure 3E** and **Supplementary Figure S1A**).

We then compared the gene expression profiles of the 5 human liver NK subsets with those of CD56^bright^ and CD56^dim^ NK cells^43^ to validate the classification of NK cells. The analysis of module scores calculated at the single-cell level^25^ Indicated that the CD56^bright^ score of L4-LrNK-XCL1 cells was similar to those of the L1-NK-CD56^bright^ cells, and the CD56^dim^ score of L5-LrNK-FCGR3A cells was similar to that of L2-NK-CD56^dim^ cells (**Figure 3F**). These results suggested the existence of 2 distinct LrNK subtypes of NK cells in the healthy human liver. The violin plots of module scores demonstrated that the L3-NK-HLA cells represented an intermediate subset between CD56^bright^ and CD56^dim^ NK cells (**Figure 3F**). Furthermore, PC1 separated the L1-NK, L2-NK, and L5-LrNK subsets, whereas PC2 separated the L3-NK and L4-LrNK subsets from the other NK cell subsets (**Figure 3G**). PCA results revealed that *COTL1* (encoding a specific actin-binding protein), *GNLY*, and *XCL1* were drivers for the L1-NK cells; cytotoxicity-associated *GZMB*, *GZMH*, and *FGFBP2* (fibroblast growth factor family member) genes were drivers for L2-NK cells; *CRTAM* and *IRF8* were drivers for the L3-NK-HLA cluster; *HLA−DRB1* and *ARRDC3* were drivers for the L4-LrNK-FCGR3A cluster; and *IL2RB*, *GIMAP7*, and *CXCR6* were drivers for the L5-LrNK-XCL1 cluster (**Figure 3G**).

The transcriptional signature of the L3-NK-HLA cells showed enrichment in genes encoding the activating receptors (*FCGR3A* and *KLRC2*) and cytotoxic cytokines (*GZMB* and *GZMH*), as well as significantly low expression of inhibitory receptors (*KLRC1* and *TIGIT*) (**Figure 3H**). In agreement with the gene expression data, Gene Ontology-based biological process enrichment analysis strongly suggested that the identified cell subsets are likely to have distinct physiological functions (**Supplementary Figure S1C**). The L3-NK-HLA cells were enriched in genes involved in positive regulation of cytokine production, leukocyte cell–cell adhesion, lymphocyte activation, and cell killing (**Figure 3I**).

### Unsupervised clustering of NK cells from patients with HCC demonstrates the absence of 2 cytotoxic NK cell subsets

**Figure 4:**
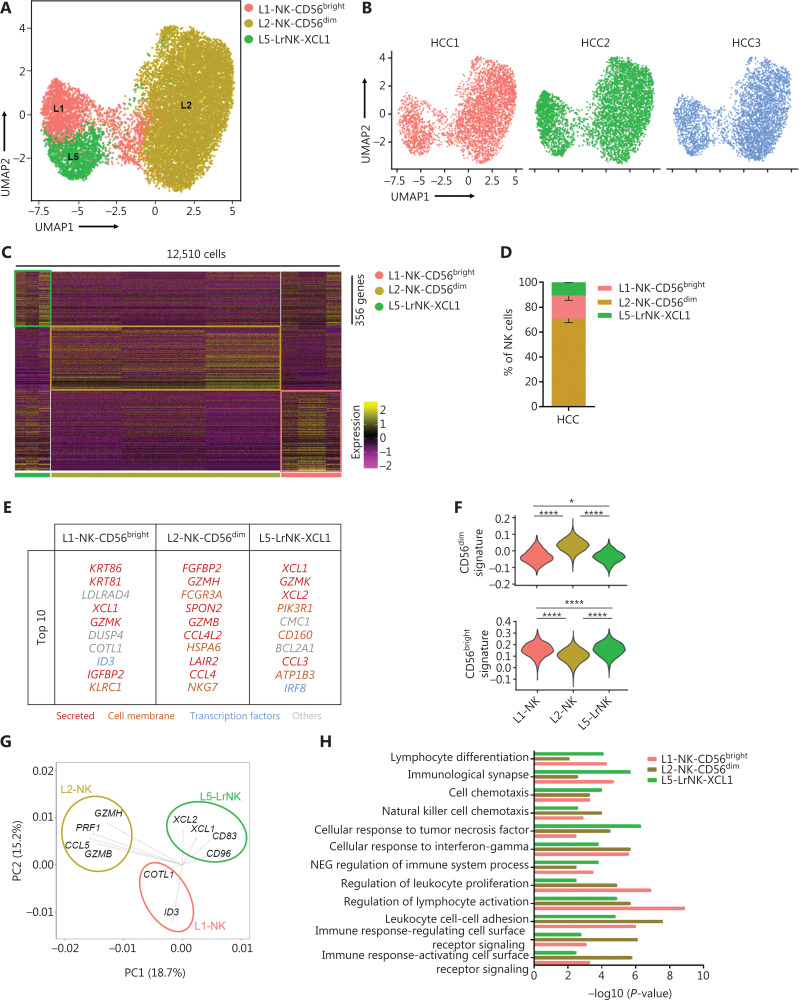
High-throughput scRNA-seq identifies 3 NK cell subsets in liver cancer. (A) UMAP projection of NK cells from 3 patients with HCC, showing 3 main subsets (in different colors). The putative functional description for each subset is based on the characteristic gene expression profiles of each subset. (B) UMAP plot of human liver NK cells from tumor infiltrating cells, colored by patient (HCC1: 3,902 cells; HCC2: 4,799 cells; and HCC3: 3,809 cells). (C) Heatmap of the 356 genes tested with a Wilcoxon rank sum test separating the 12,510 HCC-infiltrating NK cells into 3 main subsets shown in different colors. Squares identify specific transcriptomic signatures of different cell subsets. (D) The fractions of 3 subsets defined among NK cells in 3 patients with HCC across all liver NK cells. (E) Top 10 genes significantly differentially expressed among the 3 liver NK subsets. Genes are ranked by log_2_ fold-change. (F) Module scores of CD56^dim^ and CD56^bright^ gene expression programs defined by Hanna et al.^43^. Violin plots representing the distribution of module scores for CD56^dim^ (top) CD56^bright^ (bottom) for each liver NK cell subset (Kruskal-Wallis ANOVA followed by Dunn’s multiple comparisons test). *P < 0.05; ****P < 0.0001. (G) PCA for the driving genes of 3 liver NK cell subsets from HCC. (H) Selected Gene Ontology terms using genes upregulated (log_2_ fold-change > 0.25) within each subset with an adjusted *P* < 0.05.

We then performed scRNA-seq analysis to determine the characteristics of NK cells in the tumor microenvironment, by using samples from 3 treatment-naive patients with HCC. We identified 3 NK cell subsets in the liver IT tissues, according to differences in the transcriptional profiles (**Figure 4A–4C**). The L3-NK-HLA and L4-LrNK-FCGR3A subsets associated with cytotoxicity in the healthy human liver were absent in the IT tissues (**Figure 4A**). At the protein level, the average percentage of L4-LrNK-FCGR3A (CXCR6^+^CD16^+^) cells was 8.53% in the healthy liver, but their proportion in the liver cancer tissues (2.7%) was significantly lower than that in healthy liver tissues, as demonstrated by FACS (**Supplementary Figure S2A–S2C**). Furthermore, as shown by previous studies^44^, the proportion of L5-LrNK-XCL1 cells was significantly higher in the HCC tissues than the healthy liver tissues (**Figure 4D**). The top 10 differentially expressed genes in the 3 cell subsets are displayed in **Figure 4E**. The L1-NK-CD56^bright^ cells overexpressed *XCL1* and were comparable to the human CD56^bright^ NK cells. The L2-NK-CD56^dim^ cluster showed high expression of *FCGR3A* and was comparable to the human CD56^dim^ NK cells. The L5-LrNK-XCL1 cells showed high expression levels of *CD160*, *CD69*, *ICAM1*, and *TIGIT*, and were comparable to the LrNK cells. The module scores showed significant enrichment in the signature genes of CD56^dim^ NK cells for the L2-NK-CD56^dim^ cells (**Figure 4F**, top) and the signature genes of CD56^bright^ NK cells for the L1-NK-CD56^bright^ cells and L5-LrNK-XCL1 cells (**Figure 4F**, bottom). We also investigated the driver genes of the 3 NK cell subsets, according to PCA plots. L1-NK cells showed high expression of *COTL1* and *ID3* (inhibitor of DNA binding 3); L2-NK cells showed high expression of *GZMB*, *GZMH*, *PRF1*, and *CCL5*; and L5-NK cells showed high expression of *XCL1*, *XCL2*, *CD83*, and *CD96* as driver genes (**Figure 4G**). Gene Ontology biological process analysis confirmed the specific pathway enrichment of these subsets (**Figure 4H**). L1-NK-CD56^bright^ cells were associated with the regulation of lymphocyte activity. The L2-NK-CD56^dim^ cluster was associated with regulation of the immune response and activated cell surface receptor signaling. L5-LrNK-XCL1 cells were associated with the cellular response to tumor necrosis factor and the immunological synapse. In summary, the composition of the NK cell subsets was significantly altered in the HCC microenvironment, and the cytotoxic NK cell subsets were absent in HCC tissues.

### Peri-tumoral NK cells show greater anti-tumor activity than intra-tumoral NK cells

**Figure 5:**
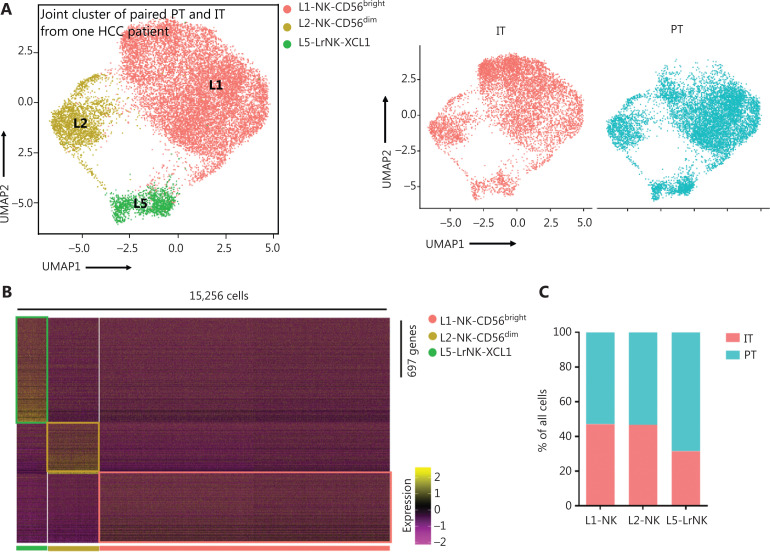

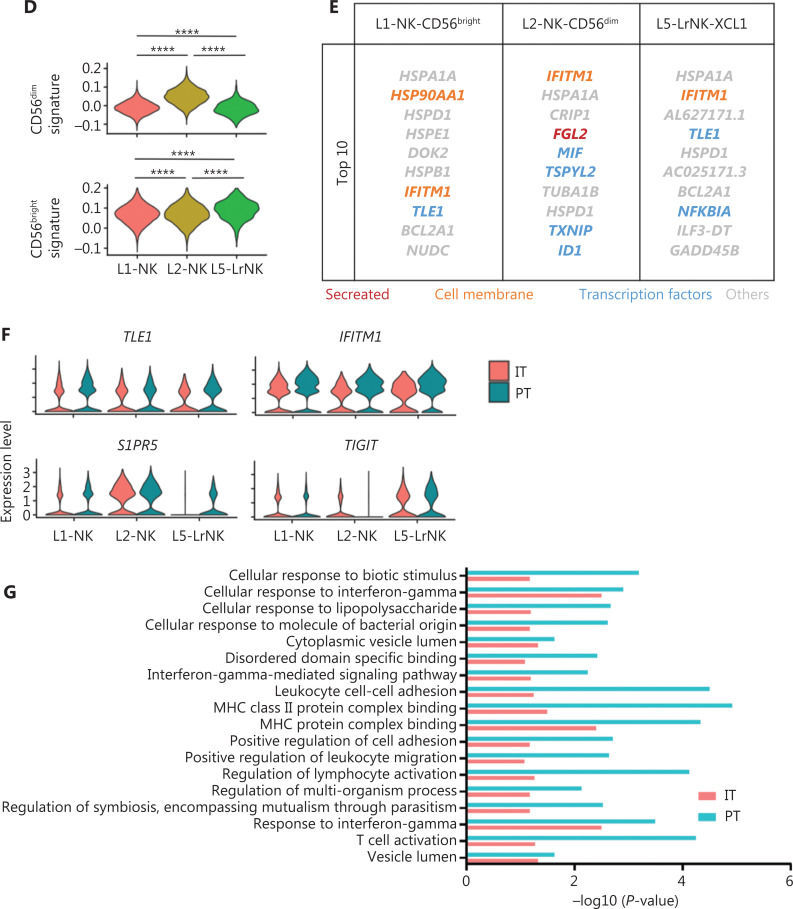
Comparison of NK cells in the IT and PT of the same patient with HCC. (A) UMAP projection of NK cells from 1 patient with HCC (ID 101; 15,256 cells), depicting 3 main subsets (shown in different colors). The functional description for each subset was determined according to gene expression profiles. UMAP plot of human liver NK cells from IT (6,976 cells) and PT (8,280 cells) of a patient with HCC. (B) Heatmap of the 697 genes tested with a Wilcoxon rank sum test separating the 15, 256 paired IT and PT NK cells into 3 main subsets (shown in different colors). Squares identify specific transcriptomic signatures for the distinct cell subsets. (C) The proportions of the 3 subsets from the IT and PT of a patient with HCC. (D) Module scores of CD56^dim^ and CD56^bright^ gene expression programs defined by Hanna et al.^43^. Violin plots representing the distribution of module scores for CD56^dim^ (top) CD56^bright^ (bottom) for each liver NK subset (Kruskal-Wallis ANOVA followed by Dunn’s multiple comparisons test). ****P < 0.0001. (E) Top 10 genes significantly overexpressed in PT compared with IT in 3 liver NK subsets. Genes are ranked by log_2_ fold-change. (F) Violin plots showing expression comparison of selected genes in NK cells. (G) Selected Gene Ontology terms using genes upregulated (log_2_ fold-change > 0.25) within each origin with an adjusted *P* < 0.05.

We then performed unsupervised hierarchical clustering analysis of the IT and PT NK cells from patient #101 with HCC to assess the heterogeneity between the NK cells in the whole liver. The clustering of paired PT and IT data revealed 3 subsets whose overall distribution was similar in the PT and IT tissues (**Figure 5A**). These findings suggested that the microenvironment of the peritumoral tissue differed from that in the healthy liver tissues. The heatmap indicated 3 distinct subsets of NK cells clearly separated on the basis of differences in gene expression patterns (**Figure 5B**). The general distribution patterns of cNK cells (L1-NK and L2-NK) were similar between the PT and IT tissues, but the proportion of LrNK NK cells (L5-LrNK) was higher in the PT tissues than the IT tissues (**Figure 5C**). This result suggested that the LrNK cells played a key role in tumor immunology. The violin plots of the module scores indicated clear enrichment in the gene signature of CD56^bright^ NK cells^43^ for the L1-NK-CD56^bright^ and L5-LrNK-XCL1 NK cells, and the enrichment in the gene signature of CD56^dim^ NK cells^43^ for the L2-NK-CD56^dim^ NK cells (**Figure 5D**). We therefore analyzed up-regulated genes among the 3 NK subsets in PT tissues compared with IT tissues to determine changes in gene expression in the tumor microenvironment (**Figure 5E**). The PT tissues showed higher expression levels of *HSPA1A*, which encodes a protein that stabilizes existing proteins and prevents protein aggregation, and *HSPD1*, which encodes a mitochondrial signaling protein that functions in the innate immune system (**Figure 5E**). Furthermore, violin plots of the NK cell clusters suggested that PT NK cells were more active than IT NK cells. For example, PT NK cells expressed higher levels of genes encoding functional molecules, such as *TLE1* (encoding a transcriptional corepressor), *IFITM1* (encoding interferon-induced transmembrane protein 1), and *S1PR5* (encoding sphingosine 1-phosphate receptor), and lower levels of the inhibitory receptor gene *TIGIT* (**Figure 5F**). Gene Ontology biological process analysis of the significantly up-regulated genes showed that the PT NK cells exhibited higher immune activity, such as responses to IFN-γ, regulation of lymphocyte activation, and leukocyte cell-cell adhesion, than the IT NK cells (**Figure 5G**). Overall, these results suggested that the PT microenvironment distinctly differed from the healthy liver microenvironment. We additionally identified 3 subsets of NK cells in both PT and IT tissues. Moreover, the NK cells from the PT tissues were more active than the NK cells from the IT tissues.

### Four prognosis-associated genes are highly expressed in paired tumor and peripheral blood samples from a patient with HCC

**Figure 6:**
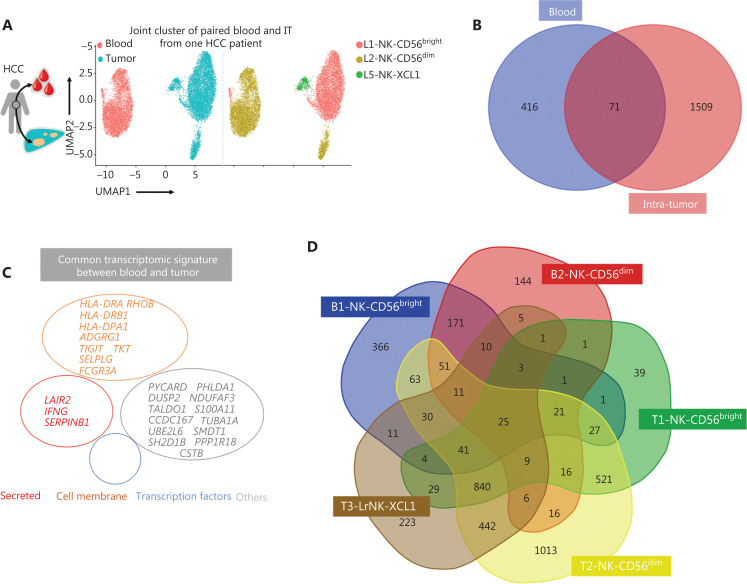

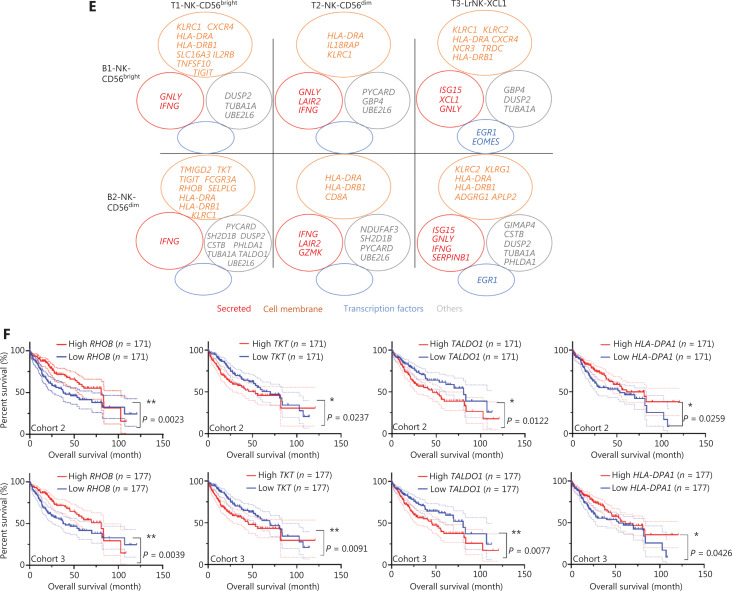
Comparison of NK cells in the tumor and peripheral blood of the same patient with HCC. (A) UMAP plot of human liver NK cells from 1 patient with HCC tumor (6,820 cells) and peripheral blood (4,756 cells), colored by cell origin (left) and subset (right). (B) Venn diagram showing common genes elevated in NK cells from tumor tissue (relative to peritumor tissue, log_2_ fold-change > 0) and those from HCC blood (relative to healthy blood, log_2_ fold-change > 0). (C) Common transcriptomic signature between genes elevated in all NK cell subsets from tumor tissue (relative to peritumor tissue, log_2_ fold-change > 0) and those from HCC blood (relative to healthy blood, log_2_ fold-change > 0). (D) Venn diagram showing common genes among NK cell subsets from HCC blood and tumor tissue (log_2_ fold-change > 0). (E) Common subset-specific immune-associated transcriptomic signatures between NK cell subsets from HCC blood and tumor tissue (log_2_ fold-change > 0). (F) Kaplan–Meier survival curves for the duration of OS in months, according to the gene expression levels of *RHOB*, *TALDO1*, *HLA-DPA1*, and *TKT* in IT from patients with HCC from cohort 2 and cohort 3 (high densities, red line; low densities, blue line) (log-rank test). *P < 0.05; **P < 0.01.

Detecting blood markers is more convenient and easier than performing histology tests in patients with cancer. Therefore, we investigated the expression levels of prognosis-associated genes in both the peripheral blood and tumor tissues to determine their clinical utility. The scRNA-seq analysis of paired blood and tumor samples from 1 patient with HCC demonstrated an absence of LrNK cells in the peripheral blood (**Figure 6A**). We then performed integrated analysis of the NK cells in blood samples from a healthy donor and a patient with HCC to identify highly expressed genes in the blood of patients with HCC (**Supplementary Figure S3A**). Venn diagram analysis demonstrated elevated expression of 71 common genes in the blood and IT tissues of patients with HCC (**Figure 6B**), including genes encoding secretory effector proteins (*LAIR2*, *IFNG*, and *SERPINB1*), membrane proteins (*TIGIT*, *TKT*, *HLA-DRA*, *RHOB*, *HLA-DRB1*, *ADGRG1*, *HLA-DPA1*, *SELPLG*, and *FCGR3A*), and other products (**Figure 6C**). Pairwise comparisons among NK cell subsets revealed 25 highly expressed common genes across the 5 NK cell subsets in the blood and tumor tissues of patients with HCC (**Figure 6D**), including genes encoding secretory effector proteins (*LAIR2*, *GNLY*, *IFNG*, *GZMK*, and *XCL1*), transcriptional regulators (*EGR1* and *EOMES*), membrane proteins (*TIGIT*, *TKT*, *HLA-DRA*, *RHOB*, *HLA-DRB1*, *ADGRG1*, *KLRC1*, *KLRG1*, *NCR3*, *FCGR3A*, and *CXCR4*), and other products (**Figure 6E**).

To explore the relationship between gene expression and patient survival, we then evaluated the potential prognostic value of these 25 highly expressed genes in the NK cells from the blood and tumor tissues of patients with HCC by analyzing TCGA data. Patients with HCC from cohort 2 (*n* = 372) and cohort 3 (*n* = 442) were divided into 2 groups on the basis of the median value of the expression of each gene. Four of 25 genes, *RHOB*, *TALDO1*, *HLA-DPA1*, and *TKT*, were associated with the prognosis of patients with HCC from both cohorts (**Figure 6F**). Higher expression of *RHOB* and *HLA-DPA1* and lower expression of *TALDO1* and *TKT* in tumors correlated with significantly longer overall survival (OS) in patients with HCC (**Figure 6F**). Prognostic value of *CCDC167*, *TUBA1A*, *SH2D1B*, and *S100A11* was found in only cohort 3 and not in cohort 2 (**Supplementary Figure S3B**). Furthermore, we observed overexpression of *RHOB* and *HLA-DPA1* and significant downregulation of *TKT* and *S100A11* in the L3-NK and L4-LrNK subsets, which were identified only in healthy liver tissues but were absent in the HCC tissues (**Supplementary Figure S3C**). We further analyzed the correlation between the expression levels of *TKT*, *RHOB*, *TALDO1*, and *HLA-DPA1* genes and the survival of patients with HCC, on the basis of NK cell infiltration into the tumor tissues (**Supplementary Figure S4**), as previously described^45^. The patients with HCC in TCGA database were classified according to the expression levels of *CD56* and related genes (higher or lower than median expression value in all patients). Then the survival of patients with high or low expression of *CD56* and the corresponding genes such as *TKT*, *RHOB*, *TALDO1*, and *HLA-DPA1* were evaluated. Low *TKT* mRNA levels were associated with significantly higher OS in patients with HCC and a higher degree of NK cell infiltration in tumors, according to *CD56* expression. Low *TALDO1* mRNA levels were associated with longer OS in patients with HCC and a lower degree of NK cell infiltration in tumors. *RHOB* mRNA levels were associated with survival outcomes of patients with HCC regardless of the degree of NK cell infiltration in the tumors. However, *HLA-DPA1* mRNA levels were not associated with the survival outcomes of patients with HCC after the patients were classified according to *CD56* expression (**Supplementary Figure S4**). In summary, our data demonstrated that the expression of these 4 genes in the peripheral blood of patients with HCC can be used in clinical settings to predict HCC prognosis, because the results are consistent with the expression in tumor tissues.

## Discussion

Single-cell RNA sequencing is used to investigate cellular heterogeneity in the human tissue microenvironment^46,47^. In humans, scRNA studies have reported whole liver cell diversity^29,48^, heterogeneity of liver-resident immune cells^30^, the landscape and dynamics of the immune cells in HCC tissues^32^, and the liver microenvironment in early relapsing HCC^31^. However, these studies have focused on the global immune system in the liver but have not characterized the heterogeneity of NK cells. For example, an scRNA-seq study on healthy human liver cells by Aizarani et al.^48^ has identified only 1 NK cell subset. Another scRNA-seq study on healthy liver-resident CD45^+^ immune cells by Zhao et al.^30^ has classified NK cells into cNK and LrNK cells. An scRNA-seq study of liver transplantation samples by Wang et al.^49^ has subdivided the liver NK cell population into 4 subsets according to the top 5 highly expressed genes but has not described their functional characteristics. In this study, we analyzed 46,726 liver-resident NK cells from 9 samples, including NK cells from healthy livers (*n* = 3), HCC tumors (*n* = 4), HCC tumor-adjacent liver tissues (*n* = 1), and blood samples (*n* = 1), to define the heterogeneity of NK cells in the human liver. Our results demonstrated distinct characteristics between liver NK cells and NK cells from the spleen and peripheral blood. The unbiased clustering of single cell sequencing results or the liver NK cell samples identified 5 distinct clusters in the healthy liver (L1, L2, L3, L4, and L5) and 3 clusters in the peritumoral and intratumoral (L1, L2, and L5) liver tissues from patients with HCC. Moreover, the L2 and L5 subsets were significantly elevated in the liver cancer tissues. In previous studies, we have demonstrated that high expression levels of NKG2A^50^ and CD96^51^ proteins in the NK cells of HCC tissues are associated with NK cell exhaustion as well as poor prognosis. A recent study has shown that high expression of the TIGIT and TIM3 proteins in the NK cells of patients with liver cancer correlates with NK cell exhaustion and disease progression^52^. Therefore, we analyzed these markers associated with NK cell exhaustion. Our results showed high expression of the inhibitory receptor genes *NKG2A*, *TIGIT*, *TIM3*, and *CD96* in the L1-NK-CD56^bright^ subset; high expression of the inhibitory receptor genes *TIM3* and *CD96* in the L2-NK-CD56^dim^ subset; and high expression of the inhibitory receptor genes *KLRC1* and *TIGIT* in the L5-LrNK-XCL1 subset (data not shown). In addition, NK cells were more active in the peritumor tissues than the intratumor tissues of patients with HCC. Moreover, we identified 4 commonly overexpressed genes in the peripheral blood and tumor tissues of patients with HCC that correlated with prognosis.

The cell surface markers of human LrNK cells have not been clearly described, in contrast to murine LrNK cells (CD49a^+^DX5^−^)^14^. Several studies have identified CD16^neg^ NK cells as human LrNK cells^15–17,53^. However, in this study, we identified a subset of CD16^pos^ LrNK cells (L4-LrNK-FCGR3A) in the healthy adult liver that showed high expression of cytotoxicity-associated genes, such as *GNLY*, *FCGR3A*, *GZMB*, *GZMH*, and the tissue residency-associated gene *CXCR6*. The L5-LrNK-XCL1 subset showed low expression of *KLRC2* (activating receptor gene) and high expression of *KLRC1* and *TIGIT* (inhibitory receptor genes). These results suggested that the L4-LrNK-FCGR3A subset was associated with strong cytotoxic functions, whereas the L5-LrNK-XCL1 subset was associated with non-cytotoxic functions. Furthermore, our findings suggested that the L5-LrNK-XCL1 subset played a major role in regulating the proliferation and differentiation of lymphocytes. The L4 and L5 liver-resident NK cell subsets were present in healthy liver tissues, but the L4 subset was absent in HCC tissues. These results suggested that in liver cancer tissues, the absence of the L4 subset of NK cells affects the immunosurveillance and cytotoxicity of HCC cells.

A transitional NK cluster with intermediate expression levels of the signature genes in CD56^bright^ or CD56^dim^CD57^+^ NK cells has been observed in human bone marrow and blood^23^. In this study, we identified the intermediate subtype of NK cells (L3-NK-HLA) with a phenotype between those of the CD56^bright^ and CD56^dim^ NK cell populations in healthy liver samples. In comparison with the CD56^bright^ NK cells and CD56^dim^ NK cells, the L3-NK-HLA cells exhibited intermediate transcriptional characteristics, according to the module score. This cluster showed high expression of *HLA-DR*, which was associated with NK cell activity. Gene Ontology biological process analysis suggested a cytotoxic role of the L3-NK-HLA cells. The L4-LrNK-FCGR3A and L3-NK-HLA clusters were found in only healthy liver tissues and were absent in the HCC tissues. This result suggested that the L4 and L3 NK cell subsets were relatively more active and represented cytotoxic NK cells, but were absent in the tumor tissues.

We also observed that the 4 prognosis-associated genes *RHOB*, *TALDO1*, *HLA-DPA1*, and *TKT* were simultaneously elevated in the tumor and peripheral blood in patients with HCC. *HLA-DPA1* is highly associated with persistent HBV infections and related HCC tissues, according to a transcriptome-wide association study^54^. Moreover, higher *HLA-DPA1* expression in the HBV-associated HCC tissues has been found to correlate with longer OS^55^. The analysis of these 4 prognostic genes in patient blood samples may provide a more precise prognosis for patients with HCC from an immunological perspective. A combination of tumor biomarkers and immunological biomarkers can improve prognostic prediction for patients with HCC. Further comprehensive analysis is required to evaluate the clinical utility of these 4 genes. Thus, transcriptome data coupled with clinical information may provide a deeper understanding of liver-infiltrating NK cells. In conclusion, our comprehensive single cell study revealed detailed characteristics of NK cells from healthy liver and HCC tissues, including the different subtypes and unique gene signatures, and their potential roles in immunity against HCC. Our study improves understanding of NK cell heterogeneity in the liver tissues of HC donors and patients with HCC, and provides new avenues for the development of more effective immunotherapies for HCC.

## Conclusions

Our study provided a panoramic description of NK cell subsets through transcriptomic comparison of NK cells from blood, healthy liver, HCC tumor tissue, and tumor-adjacent tissue through scRNA-seq. We found that the cytotoxic NK cell subsets L3-NK-HLA and L4-LrNK-FCGR3A were absent in patients with HCC. Furthermore, we identified 4 potential non-invasive prognostic biomarkers (*RHOB*, *TALDO1*, *HLA-DPA1*, and *TKT*) for patients with HCC. The findings improve the understanding of liver NK heterogeneity and may contribute to the development of immunotherapy.

## Data Availability

The accession number for the raw and processed RNA-seq data reported in this paper is GEO GSE162616. Publicly available software used in this study is listed in the Supplementary Information.
