# Supplementary material for: Panoramic comparison between NK cells in healthy and cancerous liver through single-cell RNA sequencing
Source: Cancer Biol Med. 2022 Jul 21;19(9):1334–51. doi: 10.20892/j.issn.2095-3941.2022.0050 (PMC9500227; doi:10.20892/j.issn.2095-3941.2022.0050)
Supplement: Supplementary file 1 [file cbm-19-1334-s001.pdf]

Supplementary materials

Doc S1: Supplementary materials and methods

Table S1 Clinicopathological characteristics of cohort 1.

Characteristics	scRNA-seq		FACS	
	HC	HCC	HC	HCC
Number of patients	3	4	4	6
Gender (male)	0	3 (75.0%)	3 (75.0%)	4 (66.7%)
Age (years)	51.7 ± 2.5	58.0 ± 10.5	56.8 ± 5.6	53.8 ± 12.7
Metastasis negative	-	4 (100%)	-	6 (100%)
Capsule (+)	-	4 (100%)	-	5(83.3%)
Ascites (+)	-	1(25.0%)	-	2(33.3%)
Diameter of tumor (cm)	-	5.9 ± 4.1	-	5.6 ± 2.3
HBV-positive	1 (33.3%)	4(100%)	2 (50.0%)	6 (100%)
HCV-negative	3 (100%)	4 (100%)	4 (100%)	6 (100%)
ALT (U/L)	35.3 ± 5.4	66.0 ± 7.6	46.5 ± 28.3	38.0 ± 23.1
AST (U/L)	31.3 ± 5.3	57.8 ± 12.5	31.8 ± 7.3	50.7 ± 31.2
ALB (g/L)	42.5 ± 1.3	40.0 ± 2.5	41.5 ± 1.9	41.5 ± 9.6
AFP (ng/mL)	3.4 ± 1.9	14252.2 ± 24523.5	3.9 ± 1.0	563.8 ± 986.4
TBIL (μmol/L)	12.2 ± 3.0	22.3 ± 11.4	18.0 ± 8.8	20.9 ± 8.6

Table S2 Details and characteristics of each patient enrolled in cohort 1.

scRNA-seq

Patient ID	Gender	Age	Number	ALT (IU/L)	AST (IU/L)	TBIL (µmol/L)	ALB (g/L)	AFP (ng/ml)	Tumor dia-meter (cm)	Tumor encap-sulation	Ascites	HBV	Neoplasm histologic grade	TNM stage	Lymph node meta-stasis	Tumors grow to hepatic vessels	Distant meta-stasis	Cancer type detailed	Sample type	Tumors grow to nearby organs	Date of surgery	Peritumoral tissue	Intratumoral tissue	Blood	American Joint Committee on Cancer Publication version type	HCV
101	M	50	1	64	54	21.3	43.1	0.83	3	Y	Y	Y	2-3	II	*N*	Y	*N*	Hepatocellular carcinoma	Primary	*N*	20180821	Available	Available	Available	7th	*N*
102	M	52	1	78	70	41.1	36.4	169.7	4	Y	*N*	Y	2	II	*N*	Y	*N*	Hepatocellular carcinoma	Primary	*N*	20180531	Unavailable	Available	Unavailable	7th	*N*
103	M	76	Multiple	65	39	12.1	41.5	110.3	3.5	Y	*N*	Y	3	II	*N*	*N*	*N*	Hepatocellular carcinoma	Primary	*N*	20180523	Unavailable	Available	Unavailable	7th	*N*
104	F	54	Multiple	57	68	14.6	39	56728	13	Y	*N*	Y	2	II	*N*	Y	*N*	Hepatocellular carcinoma	Primary	*N*	20180605	Unavailable	Available	Unavailable	7th	*N*
105	F	49		31	31	10	42.7	1.96				*N*						Intrahepatic cholangio-lithiasis			20181009			Unavailable		*N*
106	F	55		32	25	10.2	40.8	none				Y						Polycystic liver			20180730			Unavailable		*N*
107	F	51		43	38	16.4	43.9	4.75				*N*						Hepatic hemangioma			20181010			Unavailable		*N*

FACS:

Patient ID	Gender	Age	Number	ALT (IU/L)	AST (IU/L)	TBIL (µmol/L)	ALB (g/L)	AFP (ng/ml)	Tumor diameter (cm)	Tumor encapsulation	Ascites	HBV	Neoplasm histologic grade	TNM stage	Lymph node metastasis	Tumors grow to hepatic vessels	Distant metastasis	Cancer type detailed	Sample type	Tumors grow to nearby organs	Date of surgery	American Joint Committee on Cancer Publication version type	HCV
106	F	55		32	25	10.2	40.8	None				Y						Polycystic liver			20180730		*N*
107	F	51		43	38	16.4	43.9	4.75				*N*						Hepatic hemangioma			20181010		*N*
108	M	55		18	24	32.7	38.8	2.39				Y						Hepatic hemangioma			20190122		*N*
109	F	66		93	40	12.5	42.4	4.45				*N*						Hepatic hemangioma			20190108		*N*
110	M	66	Multiple	57	74	18.7	37.2	8.34	4	Y	*N*	Y	2	II	*N*	Y	*N*	Hepatocellular carcinoma	Primary	*N*	20180810	7th	*N*
111	M	66	Multiple	27	41	34.6	36.4	2531	10	Y	*N*	Y	1	IIIB	*N*	Y	*N*	Hepatocellular carcinoma	Primary	*N*	20181017	7th	*N*
112	M	51	Multiple	20	25	30.67	62.1	None	4.5	Y	Y	Y	2-3	II	*N*	Y	*N*	Hepatocellular carcinoma	Primary	*N*	20180806	7th	*N*
113	F	60	1	12	17	14.5	38.8	20.18	3	Y	*N*	Y	2	I	*N*	*N*	*N*	Hepatocellular carcinoma	Primary	*N*	20190110	7th	*N*
114	F	29	1	79	108	11.37	33	214.4	7	Y	*N*	Y	1	I	*N*	*N*	*N*	Hepatocellular carcinoma	Primary	*N*	20190320	7th	*N*
115	M	51	Multiple	33	39	15.77	41.6	45.32	5	*N*	Y	Y	2	II	*N*	Y	*N*	Hepatocellular carcinoma	Primary	*N*	20190403	7th	*N*

Table S3 Summary of scRNA-seq data through 10x Genomics Cell Ranger software.

Sample	Patient ID	Estimated number of cells	Mean reads per cell	Median genes per cell	Number of reads	Valid barcodes	Sequencing saturation	Q30 bases in barcode	Q30 bases in RNA read	Q30 bases in sample index	Q30 bases in UMI	Reads mapped to genome	Reads confidently mapped to genome	Reads confidently mapped to intergenic regions	Reads confidently mapped to intronic regions	Reads confidently mapped to exonic regions	Reads confidently mapped to transcriptome	Reads mapped antisense to gene	Fraction of reads in cells	Total genes detected	Median UMI counts per cell
HC1	105	14,847	22,030	1,113	327,093,834	96.90%	62.20%	97.20%	95.60%	96.80%	96.80%	97.00%	93.70%	5.60%	35.20%	53.00%	49.30%	1.50%	88.30%	21,672	3,107
HC2	107	17,880	18,729	1,126	334,892,012	97.30%	62.90%	97.20%	95.50%	96.20%	96.80%	97.00%	93.60%	5.50%	33.50%	54.60%	50.80%	1.40%	91.80%	21,107	2,759
HC3	106	16,978	22,318	1,115	378,926,046	97.10%	65.10%	97.30%	95.50%	95.40%	96.80%	96.90%	93.80%	5.50%	37.10%	51.20%	47.80%	1.40%	93.80%	20,946	2,935
HCC1	102	18,140	18,049	1,148	327,422,737	97.20%	56.00%	97.20%	95.30%	95.50%	96.80%	97.00%	94.20%	4.10%	36.70%	53.50%	49.90%	1.30%	93.60%	21,522	2,876
HCC2	103	10,264	37,916	1,275	389,173,298	97.20%	72.50%	97.20%	95.50%	96.10%	96.80%	97.20%	91.60%	4.10%	28.00%	59.50%	56.20%	1.20%	95.50%	21,707	3,481
HCC3	104	16,030	21,352	1,194	342,273,029	97.00%	56.20%	97.30%	95.60%	94.80%	96.80%	97.10%	93.80%	5.60%	36.60%	51.60%	48.10%	1.40%	92.30%	22,727	3,372
PT	101	10,135	32,261	1,060	326,968,777	96.90%	79.90%	96.80%	92.90%	95.70%	96.50%	95.60%	93.40%	3.60%	25.20%	64.60%	60.80%	1.20%	93.00%	18,885	2,888
IT	101	10,771	32,360	1,140	348,551,458	97.00%	77.30%	96.70%	93.10%	92.60%	96.40%	95.70%	93.50%	3.20%	23.60%	66.70%	62.90%	1.10%	93.10%	20,071	3,283
Blood	101	5,452	59,452	1,186	324,134,452	96.90%	87.50%	96.80%	92.60%	95.20%	96.50%	95.50%	93.50%	3.60%	21.80%	68.00%	64.20%	1.00%	92.30%	18,279	3,287

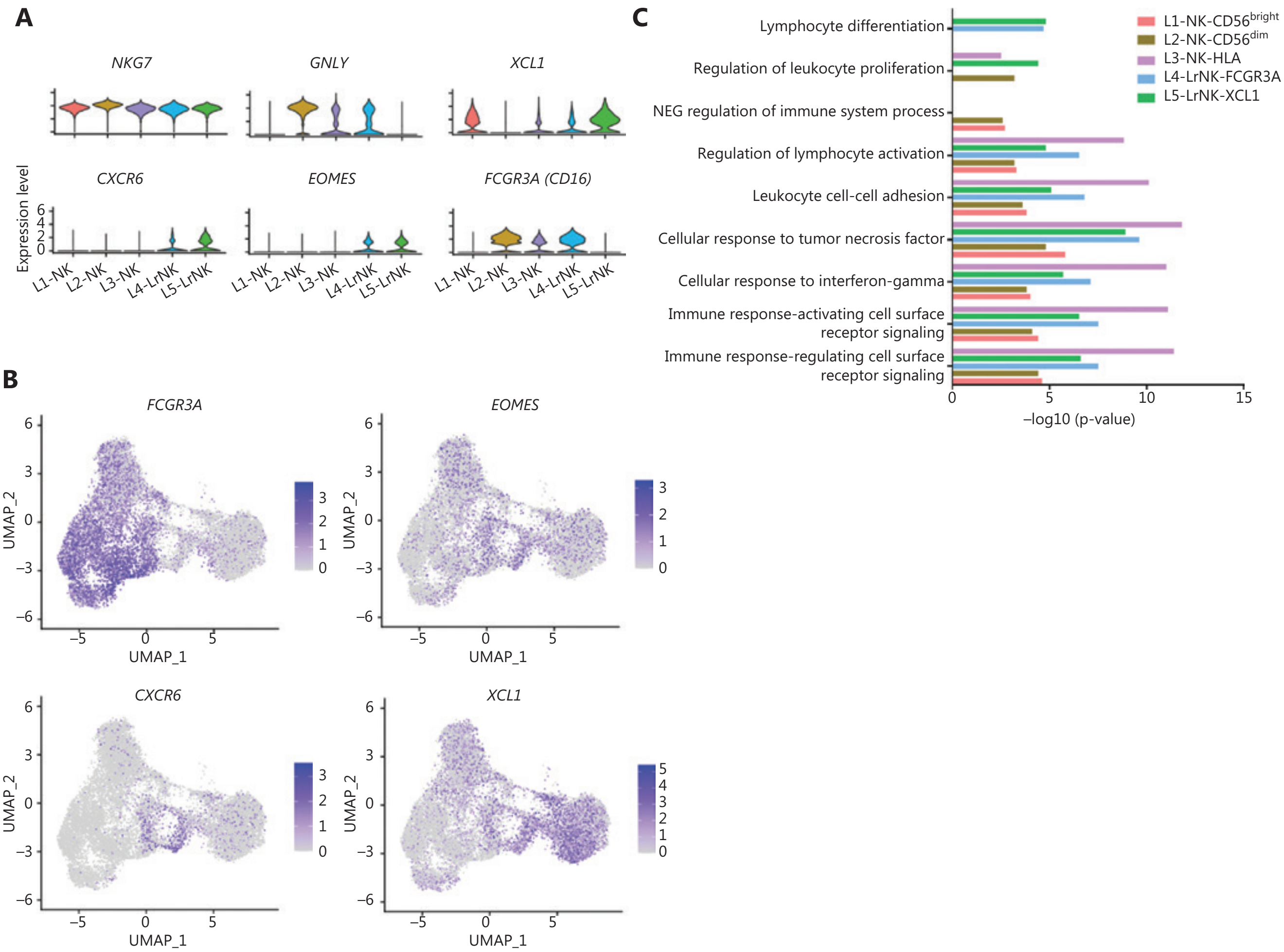

Figure S1 Cluster characterization of NK cells in the liver. (A) Violin plots showing expression comparison of selected innate lymphoid cell markers in this scRNA-seq dataset. The violin represents the probability density at each value. (B) UMAP representation of gene expression for *FCGR3A*, *EOMES*, *CXCR6*, and *XCL1*. (C) Selected Gene Ontology terms using genes upregulated ($\log_2$ fold-change > 0.25) within each subset with an adjusted $P < 0.05$.

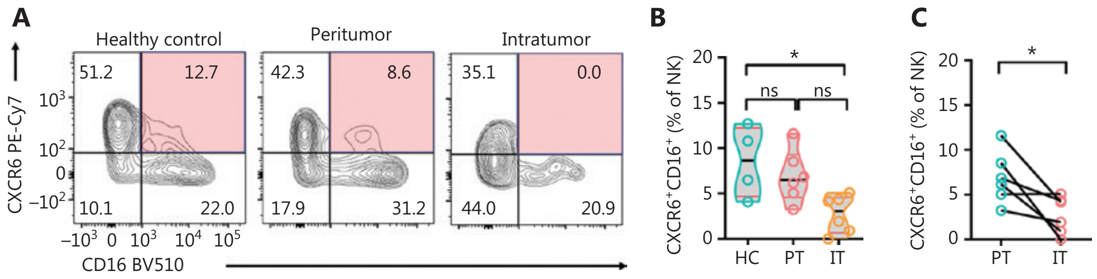

Figure S2 Frequency of CXCR6⁺CD16⁺ NK cells infiltrating healthy liver (HC, n = 4), HCC tumor-adjacent liver tissues (PT, n = 6), and HCC tumors (IT, n = 6). (A) Representative FACS plots for CXCR6⁺CD16⁺ cell expression among total NK cells from the normal liver as well as IT and PT from 1 patient with HCC. (B) Cumulative percentages of CXCR6⁺CD16⁺ NK cells in the normal liver as well as IT and PT. (C) Percentages of CXCR6⁺CD16⁺ NK cells from paired IT and PT of patients with HCC in cohort 1. One-way ANOVA test (B) and paired t test (C) were applied. Nonsignificant (ns) $P > 0.05$; *$P < 0.05$.

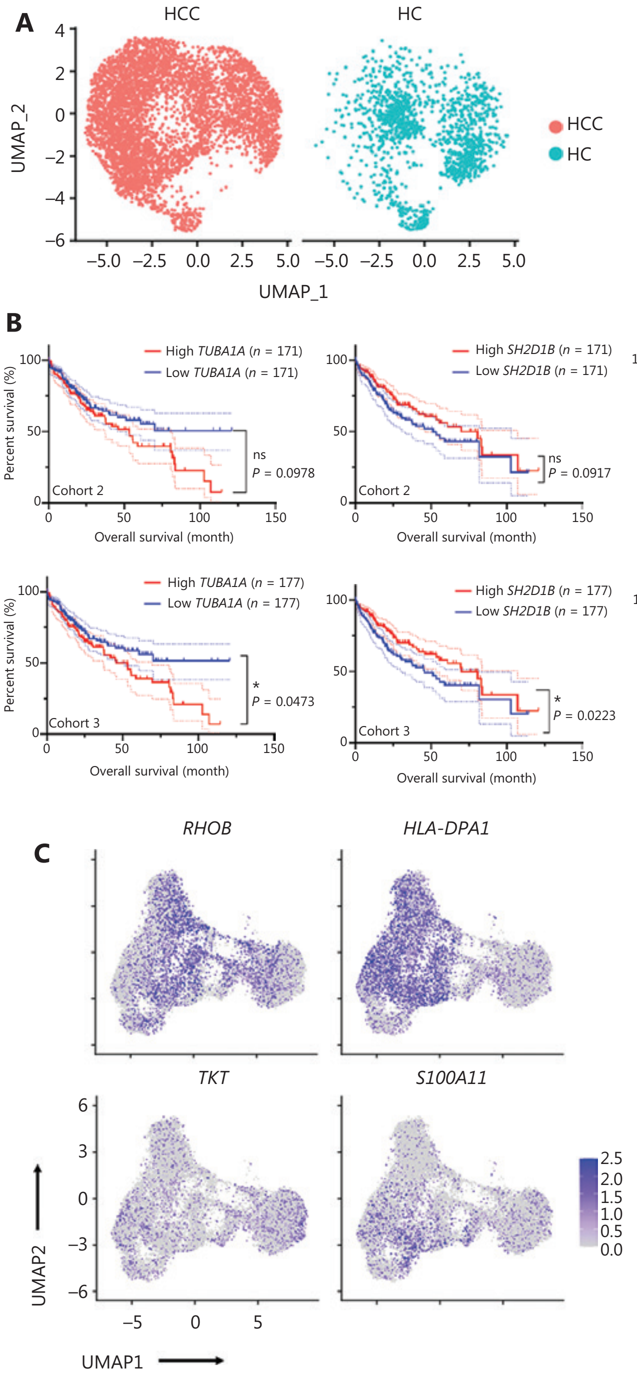

Figure S3 Cluster characterization of NK cells in the blood and IT. (A) UMAP plot of 6,182 human blood NK cells from 1 patient with HCC and a healthy donor. (B) Kaplan–Meier survival curves for the duration of overall survival (OS) in months, according to the gene expression levels of *TUBA1A*, *SH2D1B*, *S100A11*, and *CCDC167* in IT of patients with HCC from cohort 2 and cohort 3 (high densities, red line; low densities, blue line) (log-rank test). Nonsignificant (ns) $P > 0.05$; *$P < 0.05$. (C) UMAP representation of gene expression for *RHOB*, *HLA-DPA1*, *TKT*, and *S100A11*.

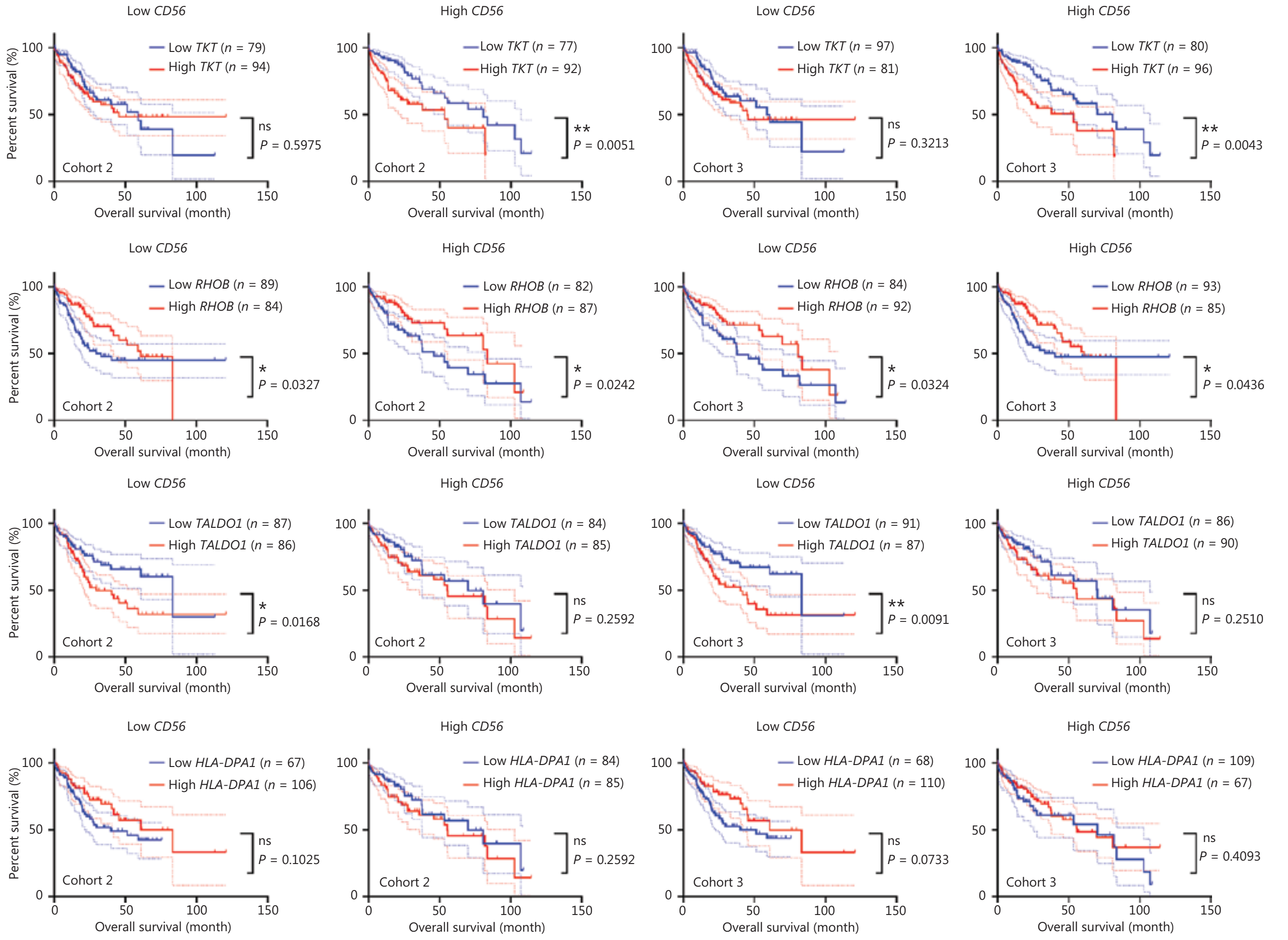

Figure S4 Correlation of *TKT*, *RHOB*, *TALDO1*, and *HLA-DPA1* expression levels depending on the calculated level of NK cell infiltration with the survival of patients with HCC. All patients in TCGA cohort 2 and cohort 3 were divided according to the expression levels of *TKT*, *RHOB*, *TALDO1*, and *HLA-DPA1* (higher or lower than the median expression value for all patients). The associations between *TKT*, *RHOB*, *TALDO1*, and *HLA-DPA1* expression level and survival are shown for patients whose tumors had higher (above the median) or lower (below the median) expression of *CD56* (log-rank test). Nonsignificant (ns) $P > 0.05$; $^{*}P < 0.05$; $^{**}P < 0.01$.
